# Management of a Partially Treated Case of Chronic Osteomyelitis of the Hard Palate and Maxillary Sinus

**DOI:** 10.7759/cureus.58983

**Published:** 2024-04-25

**Authors:** Aakanksha V Tiwari, Suwarna Dangore-Khasbage

**Affiliations:** 1 Oral Medicine and Radiology, Sharad Pawar Dental College and Hospital, Datta Meghe Institute of Higher Education and Research, Wardha, IND

**Keywords:** maxillary sinus, palatal perforation, osteomyelitis, management, hard palate

## Abstract

Osteomyelitis affects bones, including both cortex and medulla. It influences the mandible more frequently than the maxilla. Probable etiologic factors include foci of infection or trauma; however, the hematogenous spread of infection from a distant site is also a causative factor. *Staphylococcus* is the common organism involved in the causation. Clinical symptoms include signs of inflammation, pus drainage, fistulous or sinus tracts, wound disintegration, erythema, and raised local temperatures. Laboratory diagnosis with the evaluation of white blood cell count, rate of erythrocyte sedimentation, and C-reactive protein proves vitally significant. Radiographic evaluation reveals sequestra and bone destruction in the affected region. Histopathology of the lesion is confirmatory for the final diagnosis, which helps in the formulation of an appropriate management strategy. The treatment regimen usually focuses on thorough debridement of the necrotic material and an antibiotic regimen. This case report describes a male patient aged 45 years presenting with a palatal ulcer, severe halitosis, and speech difficulty. He has suffered from type II diabetes mellitus for four years. A radiological examination was carried out. Treatment was performed with surgical debridement of necrotic maxillary bone and curettage of bilateral maxillary and ethmoid sinuses under general anesthesia. Results of histopathological evaluation of the curetted material from the maxillary and ethmoid sinuses revealed osteomyelitis of the maxilla.

## Introduction

Osteomyelitis affects the bone in toto, with the common site being the mandible. Rarely maxilla can also be involved [1]. The commonest cause for the onset of the condition is odontogenic infection. *Staphylococcus aureus* is the etiologic organism in 30% to 60% of human instances of osteomyelitis, which affects the bone and bone marrow. Staphylococci are responsible for over 75% of cases. Traumatic injuries can also be considered as one of the factors responsible for infection. It can be secondary to some underlying systemic infection that tends to reach jaw bones through a hematogenous route [2]. Clinical symptoms may include pain, swelling, pus drainage, the appearance of fistulous or sinus tracts, wound disintegration, redness, and raised local temperatures [3]. Investigations may reveal alterations in leukocyte count, erythrocyte sedimentation rate, and C-reactive protein levels. The appearance of the pathogen in multiple specimens taken from deep-seated tissue is confirmed with particular staining for bacteria or fungi [4]. If certain conditions are met, acute straightforward cases may solely respond to an antimicrobial regimen, which when given for four to six weeks has an 80% success rate. On the contrary, for long-standing and implant-related osteomyelitis, antibiotic regimen alone has a lower success rate and there is a need for debridement. These debridement or revision procedures are frequently problematic since it can be difficult to determine the degree of bone debridement, and managing the resulting space with dead tissue can also necessitate complicated intrusions and take a long time to heal [5]. Despite using best practices in medical and surgical treatments, such complex situations still have a 20% probability of treatment failure [6]. This case reveals a 45-year-old male patient who reported with complaint of a palatal ulcer that was increasing in size, severe halitosis, and speech difficulty. He was operated with thorough surgical debridement. Upon histopathological evaluation of the same, the diagnosis was confirmed as osteomyelitis of the maxilla.

## Case presentation

A male patient aged 45 years, who had tested positive for COVID-19, presented to the Department of Oral Medicine with a chief complaint of painless, gradually progressing ulcer in the palate, mobility of teeth in the upper jaw, and foul smell from oral cavity for last one year.

**Figure 1:**
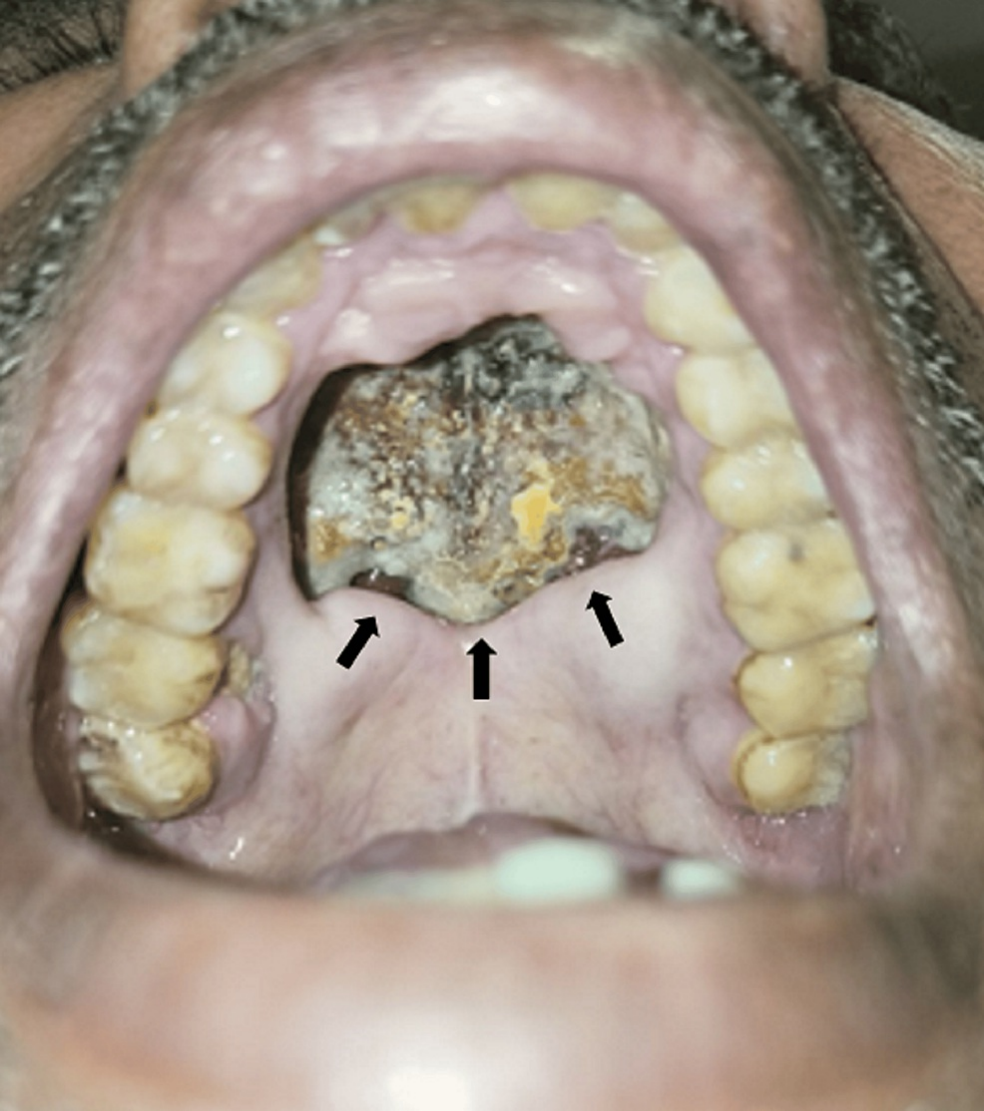
Large palatal perforation with well-defined edges involving the entire hard palate with extensive central sloughing and necrosis

History of the presenting illness revealed that he was diagnosed with COVID-19 infection two years back. After three months of infection, he noticed an ulcer in the hard palate that was small in size at initiation but gradually became larger to a size of 6 x 6 cm approximately. The patient had a history of severe halitosis and nasal twang in speech. He also experienced regurgitation of fluids from the nasal cavity. He had a known case of type II diabetes mellitus for the past four years, and there was no other significant history of hypertension, asthma, or any other blood disorder. The patient reported to a nearby physician for the same, and based on clinical features, he was diagnosed with mucormycosis of the hard palate secondary to COVID-19 infection. His blood investigations revealed significantly raised blood sugar levels. However, histopathological evaluation was not performed for confirmation of the clinical diagnosis. He was administered Fungizone 3.0 mg/kg/day for 21 days. He was also prescribed the tablet Suprax 200 mg twice daily and the tablet Zerodol Serratiopeptidase 150 mg twice daily, which he continued for 15 days. Nonetheless, the patient discontinued the therapy due to financial crises and noticed an increase in the size of the palatal ulcer thereafter. In past dental history, radiographic evaluation was done. A paranasal sinus (PNS) view radiograph and computed tomography (CT) scan were performed. Extraoral examination revealed no abnormality except submandibular lymphadenopathy on the right side measuring 0.5 x 0.5 cm approximately, which was non-tender, firm, and mobile on palpation. Intraoral examination showed a large ulcer with well-defined sharp edges in the hard palate of size approximately 6 x 6 cm. Extensive sloughing and necrotic bone were evident in the floor of the palatal ulcer but there was no apparent discharge from the ulcer. An isolated area of ulceration and sloughing was also seen in teeth 16 and 17 region of size 1 x 1 cm (Figure 1). On palpation, the maxillary dentoalveolar segment was tender and mobile.

**Figure 2:**
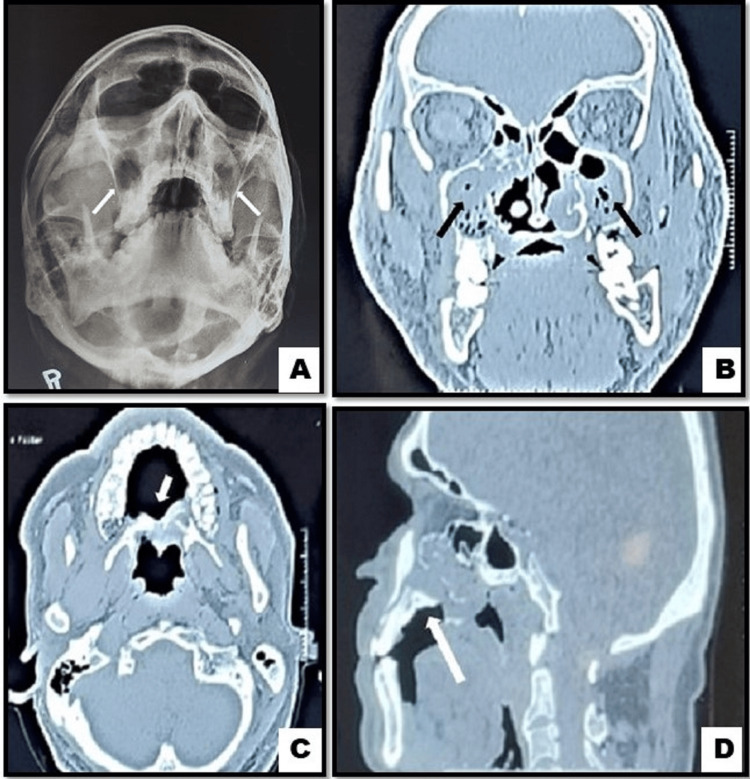
(A) Paranasal sinus radiograph; (B) Coronal section of computed tomography scan; (C) Axial section of computed tomography scan; (D) Saggital section of computed tomography scan

PNS radiograph showed evidence of haziness on the left side of the maxillary sinus. The bony margin was intact, which suggested maxillary sinusitis on the left side (Figure 2A). The coronal section of the CT scan revealed untraceable continuity of the hard palate. Radioopacity was seen in the bilateral maxillary sinus and nasal cavity suggestive of rhino-antral infective pathology leading to palatal perforation (Figure 2B). The axial section revealed discontinuity in the hard palate suggestive of palatal perforation (Figure 2C). The sagittal section showed discontinuity in the hard palate, suggesting palatal perforation (Figure 2D).

CT scan showed extensive mottled density lesion filling bilateral maxillary sinuses with an associated breach in medial and anterior walls of bilateral maxillary sinuses. There was partial destruction with mucosal thickening of the bony hard palate and alveolar ridge of the maxilla. The nasal septum was deviated to the right with soft tissue thickening. Mucosal thickening of bilateral ethmoidal, frontal, and sphenoidal sinuses was evident. R/S/O infective etiology, ? mucormycosis. Osteomyelitis of the maxilla and aspergillosis of the maxilla were considered in the differential diagnosis.

**Figure 3:**
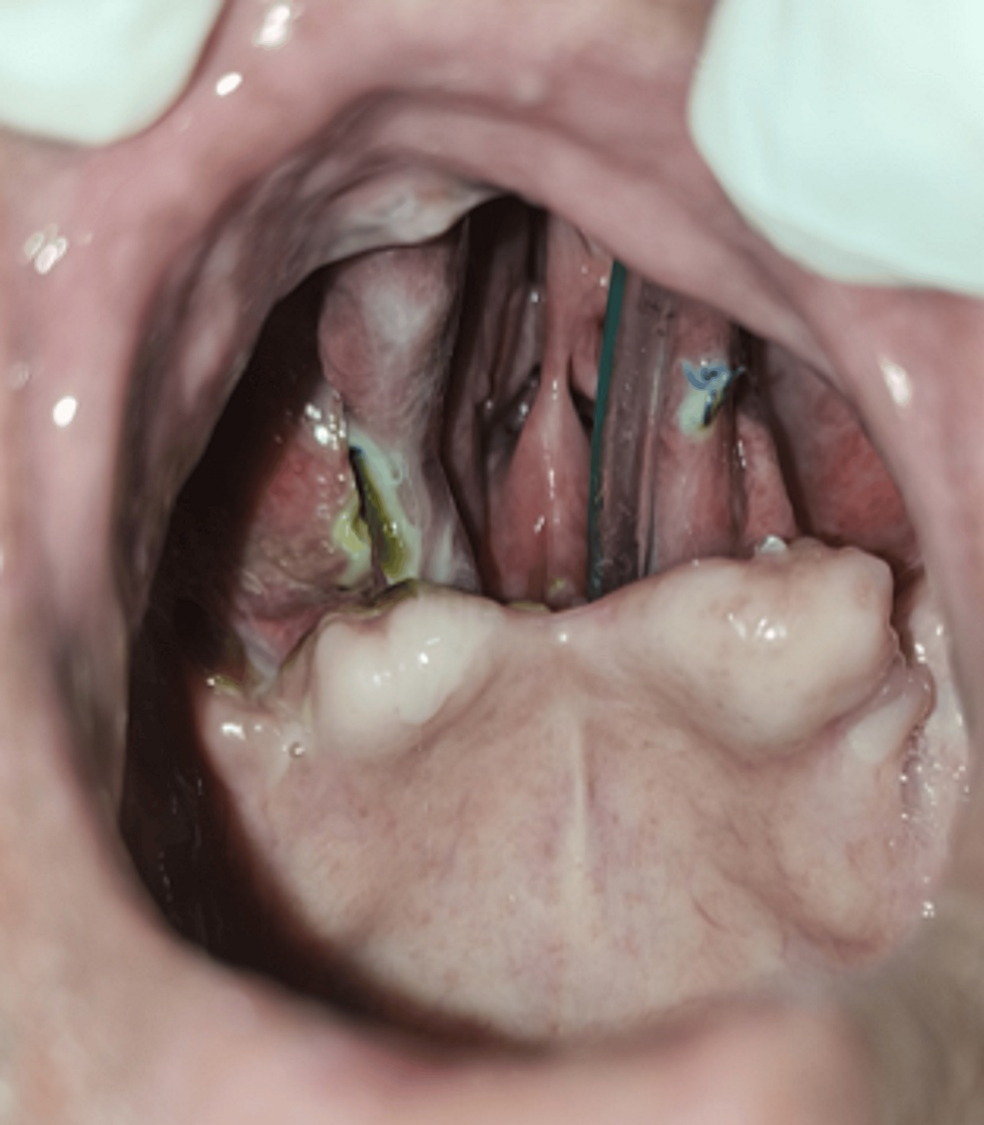
Postoperative intraoral image showing a surgical defect in the maxilla

The patient was operated on with surgical debridement of necrotic maxillary bone and curettage of bilateral maxillary and ethmoid sinuses under general anesthesia. Figure 3 shows a surgical defect in the hard palate extending into maxillary sinuses bilaterally.

**Figure 4:**
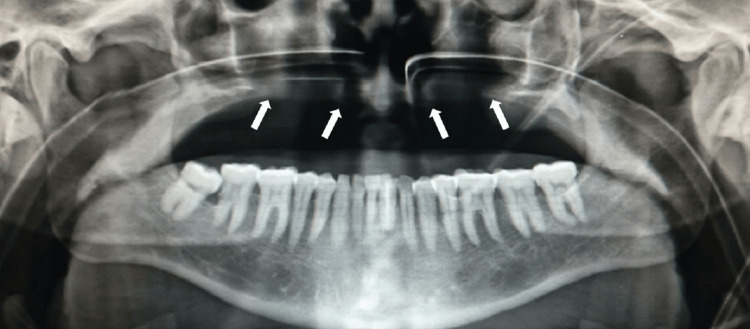
Postoperative orthopantomogram showing a surgical defect in the maxilla with loss of cortication of floor and anteromedial walls of bilateral maxillary sinuses

Postoperative orthopantomogram revealed a surgical defect in the maxillary arch with loss of cortication of inferior and anteromedial walls of bilateral maxillary sinuses (Figure 4).

Histopathological evaluation of the curetted material from the maxillary and ethmoid sinuses suggested evidence of respiratory epithelial lining with focal squamous metaplasia. The underlying tissue showed extensive areas of necrosis with foci of calcification and hemorrhages, concluding as osteomyelitis of the maxilla. Further, when the patient was recalled for postoperative evaluation, he appeared to be quite fit, hence he was advised to consult the prosthodontics department for initiation of the maxillofacial prosthesis in order to restore the function. Impression of the surgical site was taken and a temporary obturator was given to prevent nasal regurgitation. Antidiabetic medication was prescribed on discharge. The patient was advised to follow up after each month to keep a check on healing and diabetes control.

## Discussion

Osteomyelitis is an inflammatory condition affecting bones. The mandible is commonly affected in the head and neck region. Maxilla is rarely involved due to the rich blood supply from the anastomosing loop of the internal maxillary artery [7]. The most common etiology is thought to be foci of infection in the form of carious tooth or periodontal infection. Often microbes responsible for osteomyelitis include various gram-positive and gram-negative bacteria. It can be sequelae of mycotic infections like mucormycosis. The severe acute respiratory syndrome coronavirus 2 (SARS-CoV-2) is the infectious agent that causes the highly contagious COVID-19. With over six million deaths globally, COVID-19 has had a devastating impact on the planet. It has become the most significant worldwide health emergency since the 1918 influenza epidemic. COVID-19-associated mucormycosis (CAM) has been reported in individuals treated with incorrect doses of glucocorticoids, who had poorly managed diabetes, primarily rhino-orbito-cerebral mucormycosis (ROCM) [8]. Mendhe et al. described fungal osteomyelitis of the maxilla in a young male patient who had a known case of chronic type II diabetes mellitus. The patient presented with diffuse extraoral swelling on the right malar region and diffuse swelling of gingiva in the right maxillary alveolar region [9].

There are two main types of osteomyelitis: suppurative and nonsuppurative. Nonsuppurative osteomyelitis is a chronic condition with an unclear origin that does not rule out the existence of the pathogen, whereas suppurative osteomyelitis chiefly results from infection in a tooth and is defined by the purulent discharge, fistulous tract, and dead bone formation. Macbeth classified maxillary osteomyelitis into three categories: odontogenic (root sepsis), rhinogenic (spread of infection from maxillary sinus and postoperative rhinogenic instances), and traumatic (following surgical intervention or injury with the primary site of infection being maxillary sinus, odontogenic cause, or lacrimal sac) [10]. The clinical presentation, radiographic characteristics, culture, and histopathologic testing are used to make the diagnosis. Conventional radiography, CT scans, positron emission tomography/computed tomography (PET/CT) scans, cone-beam computed tomography (CBCT) scans, laser Doppler flowmetry, magnetic resonance imaging, and nuclear scans are examples of imaging modalities. The majority of the radiographic characteristics associated with chronic osteomyelitis are a "moth-eaten" appearance brought on by the expansion of medullary gaps and Volkman's canal widening as a result of bone lysis and replacement with granulation tissue. Occasionally, bone is destroyed, resulting in sequestra - islands of dead bone - and reactive subperiosteal deposition of new bone, which forms an involucrum [7].

Osteomyelitis is treated with a variety of techniques, ranging from straightforward noninvasive methods to more intrusive radical treatments. Treatment targeted towards involved microorganisms forms the mainstay. In the case of fungal etiology, antimycotic agents like amphotericin B are known to reduce the mortality rate by a certain extent [11]. Pardiwala et al. in a systematic review suggested that posaconazole suspension has been utilized as salvage therapy for maxillary mucormycosis in clinical practice and has demonstrated satisfactory efficacy in many cases, which also suggests an encouraging potential of the novel formulations featuring increased drug exposures. Hence, it can be used as a single drug regimen for the management of maxillary mucormycosis with a better survival rate and satisfactory outcomes in both immunocompetent as well as immunocompromised patients [12].

In all cases, treatment can involve conservative excision of the diseased bone with sufficient clearance; however, in cases of osteoradionecrosis (ORN)-related osteomyelitis, resection must be five times more aggressive. In a systematic review of the treatment of oromaxillofacial invasive mucormycosis, Li et al. conducted a retrospective analysis of the management approaches available for the condition and their outcomes. The analysis included noninvasive management with antifungal drugs and invasive procedures like surgical debridement for more extensive lesions [13]. The pathology inside the maxillary sinus and the unhealthy bone above the zygoma can both be cleared with a nasal endoscopy. Reconstruction of surgical defect created after debridement needs to be closed with preferably a pedicled flap rather than a free flap. To optimize tissue transfer outcomes, a plethora of perioperative strategies and considerations are employed. These strategies include close observation postoperatively of patients' hemodynamic physiology, strict blood sugar control, nutritional support, technique of surgery intraoperatively, and recognition of psychological and psychiatric factors [14]. Adjuvant hyperbaric oxygen therapy has been shown to have a direct antifungal effect and to be an efficacious method in certain regimens, particularly for mucormycosis patients caused by diabetic ketoacidosis [15].

Because of the intricate architecture and significant functional and aesthetic value of the maxillary region, reconstructing the maxilla following tumor removal is a difficult procedure. Depending on the degree of the resection as well as the specific needs and goals of the patient, this procedure may combine reconstructive surgery, prosthetic devices, and/or implant-supported restorations in an effort to restore function, aesthetics, and quality of life for patients. The gold standard for maxillary reconstruction currently consists of free flaps from the iliac crest, scapular, or fibula. These flaps allow enough bone to be brought to the recipient site for dental implant placement, which can be done concurrently with the reconstruction, and they allow for the creation of a stable division between the mouth and the nasal and paranasal sinuses. Since one of the main factors lowering these patients' quality of life is the loss of dental components, implant-prosthetic rehabilitation ought to be taken into consideration as one of the treatment's goals [16].

De Riu et al. in 2023 reported a case of entire maxillectomy restoration using a temporal muscle flap and a specially-made subperiosteal implant in a patient who was not able to have a free flap used for bone reconstruction. This method corrected an oroantral connection with a small palatal obturator and restored the boundary between the mouth and the oral cavity, giving the dental prosthesis a strong anchor. Additionally, the central part of the face received the appropriate soft tissue support from the subperiosteal implant. For patients unable to undergo free bone flap reconstructions, subperiosteal implants can be safe and useful even in primary maxillary reconstructions [17]. 

Our patient had progressed past the noninvasive conservative therapy stage as the treatment was discontinued. It led to rapid spread of infection causing palatal perforation along with invasion of maxillary sinuses. The severe necrosis of the maxillary bone was discovered by a CT scan, indicating that the affected area was ischemic and avascular. With the aid of an endoscope, the necrotic maxilla and mucosa were severely removed, and the disease was completely cleared.

## Conclusions

Osteomyelitis of the maxilla is a rare occurrence and can have detrimental effects on surrounding structures as it can spread to involve the maxillary sinus from where it can reach the cranial cavity and can thus become fatal. Etiologic factors can be infectious bacterial species and sometimes fungal infections like mucormycosis. COVID-19 infection is known to cause mucormycosis, which may cause perforation when it involves the hard palate, and clinical presentation may represent osteomyelitis. Early recognition and prompt intervention are of utmost importance to limit the spread of the disease. Stress must be imposed on educating the patient about the severity and fatal outcome of the infection. Also, early noninvasive intervention needs to be followed appropriately by the patient without any interruption so as to receive the complete benefit of treatment. A partially treated case as mentioned above can become worse and may lead to diminished prognosis due to invasion of surrounding structures. Such partially treated cases progress past noninvasive treatment options and require aggressive management like surgical debridement of necrotic bone.
